# Genetic by environment interactions affect plant–soil linkages

**DOI:** 10.1002/ece3.618

**Published:** 2013-06-12

**Authors:** Clara C Pregitzer, Joseph K Bailey, Jennifer A Schweitzer

**Affiliations:** Department of Ecology and Evolutionary Biology, University of TennesseeKnoxville, Tennessee

**Keywords:** Above- and belowground linkages, community and ecosystem genetics, feedbacks, genetic × environment interactions, nutrient cycling, *Populus*

## Abstract

The role of plant intraspecific variation in plant–soil linkages is poorly understood, especially in the context of natural environmental variation, but has important implications in evolutionary ecology. We utilized three 18- to 21-year-old common gardens across an elevational gradient, planted with replicates of five *Populus angustifolia* genotypes each, to address the hypothesis that tree genotype (G), environment (E), and G × E interactions would affect soil carbon and nitrogen dynamics beneath individual trees. We found that soil nitrogen and carbon varied by over 50% and 62%, respectively, across all common garden environments. We found that plant leaf litter (but not root) traits vary by genotype and environment while soil nutrient pools demonstrated genotype, environment, and sometimes G × E interactions, while process rates (net N mineralization and net nitrification) demonstrated G × E interactions. Plasticity in tree growth and litter chemistry was significantly related to the variation in soil nutrient pools and processes across environments, reflecting tight plant–soil linkages. These data overall suggest that plant genetic variation can have differential affects on carbon storage and nitrogen cycling, with implications for understanding the role of genetic variation in plant–soil feedback as well as management plans for conservation and restoration of forest habitats with a changing climate.

## Introduction

While it is recognized that biodiversity in forested ecosystems can maintain ecosystem processes such as aboveground productivity, decomposition, nutrient cycling, and carbon (C) sequestration (Hooper 2012; Tilman et al. 2012), only recently have empirical studies shown that intraspecific genetic diversity may be just as important to the maintenance of these processes (Madritch and Hunter 2002, 2003; Schweitzer et al. 2004, 2012a; Crutsinger et al. 2006, 2008; Lojewski et al. 2009, 2012; Clark 2010). Multiple studies demonstrate that plant genetic variation can regulate species interactions, community structure, and ecosystem processes (see reviews in Johnson and Stinchcombe 2007; Hughes et al. 2008; Bailey et al. 2009; Schweitzer et al. 2012b). For example, work in both the field and in common gardens in multiple systems has shown that phenotypic variation among individual plant genotypes or populations can influence soil microbial communities and nutrient mineralization processes as well as overall pools and fluxes of soil nutrients (Madritch and Hunter 2002; Bartelt-Ryser et al. 2005; Schweitzer et al. 2005, 2008, 2011; Madritch et al. 2006, 2009; Madritch and Lindroth 2011). Mechanistically, these plant–soil linkages (defined as the reciprocal effects of plants on soils; Bardgett and Wardle 2010) are often mediated by plant growth and chemical traits that influence soil communities and the nutrient processes they regulate in soils (i.e., decomposition and mineralization). The expression of functional plant traits that may ultimately affect ecosystem processes is known to vary intraspecifically (Cianciaruso et al. 2009; Albert et al. 2010; de Bello et al. 2010) as well as vary depending on abiotic and biotic environmental factors, it makes it important to understand the interplay of genetic and environmental variation on ecosystem level processes. However, direct measures of the strength of an ecosystem level response to genetic variation across natural environmental gradients in the field are rare (Madritch et al. 2009).

Complex phenotypes – a phenotype influenced by many gene loci, and in this case likely by multiple soil organisms (Wade 2007; Bailey et al. 2009; Genung et al. 2011) – are thought to be strongly influenced by environmental variability and genetic by environment (G × E) interactions. When G × E interactions have been examined, studies indicate that abiotic factors such as site/spatial variation differences (Johnson and Agrawal 2005; Smith et al. 2010; Tack et al. 2010), nutrient addition (Madritch et al. 2006; Boydon et al. 2008; Rowntree et al. 2010; Tomas-Nash et al. 2011), as well as biotic factors such as genotypic diversity (Crutsinger et al. 2006; Johnson et al. 2006; Madritch et al. 2006; Genung et al. 2012) and herbivory (Schweitzer et al. 2005; Madritch et al. 2007) can all influence the community and ecosystem level impacts of plant intraspecific genetic variation to varying degrees (weakly to strongly). For example, Madritch et al. (2006) found that environment moderated the effects of genotype on leaf litter decomposition and nutrient release dynamics when *Populus tremuloides* genotypes were grown under different nutrient treatments (i.e., G × E interaction occurred). Moreover, they found that the nutrient treatment environment affect was stronger than both genotype and genotypic mixtures. Understanding the role of genetic versus environmental variation in regulating ecosystem processes is important in understanding ecosystem function over time and across landscapes (Madritch et al. 2009; Fischer et al. 2010). Utilizing natural environmental variation such as temperature across elevation broadens our understanding of the ecological importance of genetic variance in ecological time frames that may have evolutionary implications (Pregitzer et al. 2010; Schweitzer et al. 2012b).

The dynamics of C and N processing in soils may be especially sensitive to natural environmental variation across elevational gradients, as multiple factors across elevations (e.g., plant phenotype, soil communities, soil moisture, temperature, pH, soil texture) may all interact to directly and indirectly influence soil processes. For example, elevational gradients result in variation in temperature, precipitation, and edaphic factors that may influence soil microbial communities, enzymatic processes, and mineralization (Waldrop and Firestone 2006; Bryant et al. 2008). Ecosystem processes, such as soil C and nitrogen (N) dynamics, can be directly linked to plant phenotypes, across plant genotypes and environments and one would expect to see phenotypic plasticity and G × E interactions in both plant phenotypes and associated soil nutrient dynamics (sensu Conner and Hartl 2004; Johnson and Agrawal 2005; Bangert et al. 2006; Bailey et al. 2009). Utilizing three 18- to 21-year-old common gardens (with common genotypes of *Populus angustifolia* planted in each) across a 294 m elevational gradient, we tested the role of environment, tree genotype, and G × E on functional plant traits, soil C and N pools and annual rates of net N mineralization (N_min_) and net nitrification. Using a common garden approach with replicated *P. angustifolia* genotypes reciprocally planted across three sites allows us to explicitly examine the impacts of plant genotype and environmental variation on plant and soil processes. On the basis of previous studies we hypothesized: (1) *P. angustifolia* genotypes will vary in phenotypic traits which will influence soil C and N pools and rates of N cycling beneath their canopy; (2) the effects of genetically based plant–soil linkages will vary by environment, and (3) Changes to soil pools and processes will be linked to changes in plant phenotypes across environments. Here, we predicted that plant genetic factors, environment and G × E interactions would influence the regulation of soil nutrient pools and fluxes through environmental variation and phenotypic plasticity in plant traits. Specifically, based on previous studies (Schweitzer et al. 2004, 2008) we predict that genetic-based traits such as foliar chemistry (lignin and condensed tannins) will vary between genotypes and across common gardens and impact soil N pools and process rates, respectively.

## Materials and Methods

### Common garden environments

To determine the influence of plant genotype and environment on plant–soil linkages we utilized three common gardens, with common plant genotypes, planted at different locations along an elevational gradient in riparian areas of the Weber River in Utah. For this study, we used five replicated *P. angustifolia* genotypes which were randomly and reciprocally planted in each garden (3–5 clonal replicates of each genotype per garden, *n* = 68 total mature trees). The Ogden Nature Center garden in Ogden, UT (41.26N, 112.11W) (∼1300 m elevation) was planted in 1991, the North Pit garden, Uintah, UT (41.49N, 111.56W) (∼1384 m in elevation) was planted in 1989, and Taggart garden, Taggart, UT (41.57N, 111.19W) (∼1594 m in elevation) was planted in 1988. Each garden is within 500 m of the Weber River and all three common gardens are separated by >20 km. All common gardens are composed of trees that were propagated from local field trees of known genotype previously determined by restriction fragment length polymorphism (RFLP; Keim et al. 1989; Martinsen et al. 2001). Mean, minimum, and maximum annual precipitation and temperature patterns for each garden were compiled using weather station data (all stations <3 km from each garden site) from 1988 to 2005 (University of Utah Climate; http://climate.usurf.usu.edu/). The gardens differ in mean annual precipitation by 150 mm (448–600 mm) and mean annual temperature by 3°C (7.7–10.7°C). All soils in the gardens are alluvial, sandy-loams characterized as mesic Oxyaquic Haploxeroll (lowest elevation, Nature center), mesic Typic Haploxerolls (North Pit), and frigid Fluventic Haploxeroll (highest elevation, Taggart) (Table 1).

**Table 1:** Mean soil characteristics (pH, soil texture, total soil carbon [C] and nitrogen [N], and ratio of C to N) and tree traits (condensed tannins and lignin for leaf [lf] or root [rt] tissues and diameter at breast height [DBH]) collected from five *Populus angustifolia* genotypes (*n* = 3–5 replicates each) in three common gardens planted in northern Utah, U.S.A

Garden	Genotype^1^	pH	% Sand/Silt/Clay	Soil C	Soil N	C:N	% CT_lf_	% CT_rt_	% Lignin_lf_	% Lignin_rt_	% N_lf_	% P_lf_	DBH (cm)
**Nature center**^1^		**7.35**	**49/39/12**	**5.28**	**0.35**	**15.17**	**2.97**	**0.84**	**24.64**	**31.75**	**1.21**	**0.35**	**26.08**
	10	7.42	44/45/12	5.47	0.34	16.13	3.07	0.90	26.86	34.51	1.086	0.30	27.28
	1000	7.20	73/16/10	5.85	0.38	14.99	5.21	0.82	26.14	34.00	1.13	0.19	32.50
	1008	7.42	36/51/13	5.67	0.34	16.62	1.77	0.78	23.05	30.43	1.38	0.49	25.14
	1019	7.20	49/41/10	4.03	0.31	12.68	3.23	1.07	24.95	29.71	1.11	0.27	9.75
	1023	7.35	43/44/13	4.96	0.37	13.76	3.14	0.78	23.52	30.09	1.21	0.35	31.02
**North pit**^1^		**6.96**	**59/31/10**	**1.91**	**0.17**	**11.37**	**5.41**	**1.17**	**23.41**	**33.66**	**0.85**	**0.22**	**14.96**
	10	6.88	64/26/10	1.63	0.16	10.34	6.15	1.38	28.20	35.24	0.90	0.21	13.38
	1000	6.97	38/49/13	1.98	0.18	10.99	5.16	0.86	26.18	32.85	0.84	0.18	14.02
	1008	6.88	36/51/13	1.71	0.14	12.53	4.82	1.10	21.99	32.46	0.86	0.30	13.60
	1019	7.11	49/41/10	1.99	0.19	10.33	6.01	1.24	22.20	32.65	0.90	0.21	17.12
	1023	6.97	82/10/8	2.28	0.18	12.65	4.97	1.45	21.96	35.14	0.78	0.20	16.84
**Taggart**^1^		**7.57**	**33/49/17**	**3.83**	**0.25**	**15.58**	**3.51**	**0.93**	**18.96**	**32.38**	**0.99**	**0.27**	**19.64**
	10	7.58	34/49/16	3.97	0.26	15.27	7.46	0.87	22.34	33.14	1.06	0.29	18.36
	1000	7.52	31/51/18	3.83	0.26	14.93	2.85	0.85	19.21	32.66	1.03	0.24	17.46
	1008	7.57	20/61/19	3.74	0.22	16.80	1.95	1.10	16.70	32.11	0.89	0.30	13.25
	1019	7.56	52/32/14	3.76	0.26	14.67	2.18	0.99	18.13	32.83	0.90	0.23	24.83
	1023	7.63	29/52/19	3.86	0.24	16.47	4.26	0.89	19.19	31.20	1.11	0.28	25.18

Averages for all genotypes within each garden are in bold.

1 Mean values for garden.

### *Populus angustifolia* genotypes

To determine variation in tree phenotype and biotic inputs among genotypes across the common gardens we assessed a suite of plant growth and chemical traits collected from each tree genotype and replicate across all common gardens. These differences allowed us to determine the influence of genotype, environment, and G × E interactions on plant phenotype (expressed as tissue quality and tree diameter at breast height; DBH) as well as on soil C and N pools and process rates associated with each plant genotype. Differences in tree diameter (DBH) among *P. angustifolia* genotypes were measured in June of 2009. To determine differences in tissue quality among *P. angustifolia* genotypes across each common garden, we measured concentrations of lignin and condensed tannin (CT) on plant leaf litter and root tissues collected from each genotype. To collect leaf litter, a mesh bag was tied around one random branch from each tree before leaf senescence and the litter was then collected in early December 2008. To quantify variation in root tissue quality among genotypes and across gardens, we collected ∼10 g of fine roots (<2 mm) from beneath each tree in June 2009 (from 0 to 15 cm). The roots were gently washed in deionized water to remove soil. Both leaf litter and roots were air-dried and ground to 20 mesh on a Wiley Mill. To quantify lignin concentrations in the ground leaf litter and root tissues, we used the acid-fiber detergent method using an Ankom 200 fiber analyzer (Ankom Technology, Macedon NY); *Quercus rubrum* leaf litter was used as a standard. Condensed tannins were extracted from the ground leaf litter and root tissues with 70% acetone + 10 mmol/L ascorbic acid and then assayed using the butanol-HCl method, using purified condensed tannin from *P. angustifolia* as a standard (Hagerman and Butler 1989). Total leaf litter N and phosphorus (P) were determined by modified micro-Kjeldahl digestion (Parkinson and Allen 1975) and analyzed on a Lachat AE Flow Injection Analyzer (Lachat Instruments, Inc., Loveland, CO), using the salicylate and molybdate-ascorbic acid methods, respectively (Lachat Instruments, Inc., 1992). There was not enough root material to quantify root N and P. All tissue chemistry data are presented on a percent air-dry mass basis.

### Soil analyses

We quantified pools of soil organic C and total N as well as annual rates of net N mineralization and net nitrification in soils beneath the canopy of all replicates of tree genotypes in all gardens to determine the relative role of plant genotype, environment, and G × E interactions. Total organic C and total N were determined on air-dried soils collected from beneath each tree (June 2008) using a Thermo CHN analyzer (Thermo Electron, Milan, Italy). Field incubations were conducted beneath each replicate genotype to assess soil net N mineralization rates between genotypes and across gardens from June 2008 to June 2009 with four sequential incubations throughout the year, at the seasonal boundaries (Hart et al. 1994; Schweitzer et al. 2004). The summer (May–September) and winter (December–March) incubations lasted approximately 3.5 months while the spring (March–May) and fall (September–December) incubations lasted approximately 2.5 months. On the east and west side of each tree in the common gardens, two polycarbonate soil cores (4.8 × 15 cm) were inserted <0.25 m from the base of each tree trunk into the soil to a depth of 15 cm (0–15 cm). One of the soil cores was removed immediately and taken back to the laboratory to determine initial soil inorganic-N pools, the other soil core was left in the soil to incubate over the course of the season when relative soil moisture and temperature was similar to bulk soil averages. Upon removal from the field, soils were transported immediately to the laboratory on ice and were kept cool (∼4°C) until processing, within 36 h. Soils were sieved (<4 mm) to remove any coarse fragments and roots and one 20 mL subsample was placed in a specimen cup, and immediately extracted with 100 mL of 2 mol/L KCl. Each extract was shaken for 1 h, gravity filtered with Whatman filter paper no. 1 (first leached with deionized water and 2 mol/L KCl) and stored in a freezer at 0°C until analyzed for extractable ammonium (NH_4_^+^) and nitrate (NO_3_^−^) (Lachat AE auto-analyzer). The incubated soils were collected at the end of each season and processed in the same manner. Annual rates of N mineralization (NH_4_^+^ + NO_3_^−^) and net nitrification were calculated by summing the seasonal rates. A subsample from each soil sample from each collection date was oven dried (48 h at 105°C) to determine soil gravimetric water content. All final soil N data are presented on an oven-dry mass basis.

Additionally, two soil samples taken from beneath each tree during the June 2008 collections were pooled, air dried, and used to determine soil pH using the 0.1 mol/L CaCl method (Hendershot et al. 1993). Soil texture (i.e., particle size) was determined for each genotype (replicate samples pooled within common gardens) using the hydrometer method (Gee and Bauder 1986).

### Statistical analyses

Plant traits, soil mineral nutrients, and abiotic parameters collected from the common genotypes across gardens were analyzed using a Generalized Linear Model. Environment refers to common garden environments in all analyses. Genotype, environment, and G × E interaction were treated as fixed effects. Soil N, C, and pH were log +1 transformed to normalize the data to determine the relative influence of genotype, environment, and G × E interactions on these response variables.

Net N mineralization data were analyzed using repeated measures multivariate analysis of variance (MANOVA). Genotype, environment and G × E interaction were treated as fixed effects. Across all seasons, log +1 transformed data were used to determine the relative influence of genotype, environment, and G × E interactions. The Wilkes Lambda statistic was used to test for significance of the repeated measure tests.

To determine the potential role of plant traits as mechanisms influencing soil nutrient pools and process rates, two approaches were taken, respectively: (1) analysis of covariance (ANCOVA) models were used to determine whether genetically based plant traits consistently impacted soil nutrient pools and mineralization processes within each site; and (2) multiple regression was used to examine phenotypic correlations among genetically based plant traits and soil nutrient pools and processes (net N mineralization) across all sites along the elevation gradient. We constrained the model to those plant traits, which were known to influence soil nutrient dynamics (i.e., leaf/root lignin, leaf condensed tannins, DBH) and varied by plant genotype and common garden environment. Two-way interactions between plant traits and common garden were also included as fixed effects. We tested for colinearity among the plant traits using a multivariate correlation matrix testing all possible pairwise relationships. Tree DBH and leaf litter CT were the only response variables that were weakly correlated (*r* = −0.26) and so all plant response variables described above were included in the model. JMP 9.0 was used for all analyses (SAS Institute, Cary, NC).

## Results

### Genetic effects

**Figure 1:**
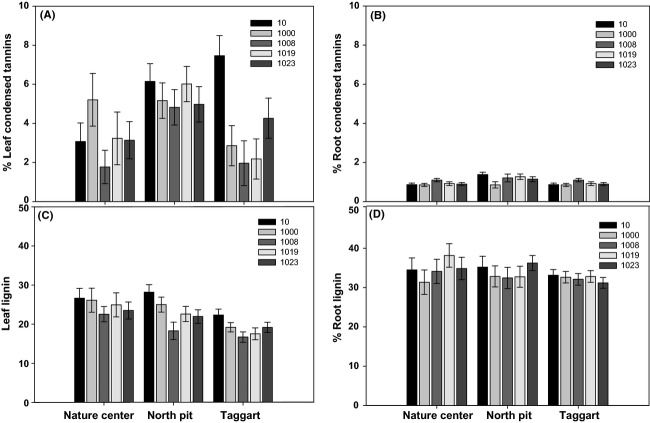
Role of plant genotype (G), environment (E), and G × E interactions on leaf litter and root secondary chemistry. (A) Mean leaf litter condensed tannins (CT), (B) root CT, (C) leaf litter lignin, and (D) root lignin, averaged from five *Populus angustifolia* genotypes reciprocally planted in three common gardens in northern Utah. (*n* = 3–5 replicates of each genotype/garden). Each bar represents a different genotype ±1 SE. See Tables 1 and 2 for statistical results.

Consistent with our hypothesis that *P. angustifolia* genotypes will vary in phenotypic traits and thus impact soil C and N dynamics we found significant differences in DBH and tissue quality among genotypes (Table 2). Leaf lignin and leaf CT were significantly different among the genotypes; across the five *P. angustifolia* genotypes leaf lignin differed between 1.3% and 11.5% and leaf CT differed between 1.3% and 5.8% (Table 2; Fig. 1). Neither root lignin nor root CT varied significantly by genotype (Table 2; Fig. 1). Leaf P content differed by plant genotype and the ratio of lignin:N was marginal (*P* = 0.06).

**Table 2:** Generalized linear model results showing χ^2^ and *P* values (below) for soil and plant traits between genotypes (G), across common garden environments (E), and G × E interactions

	Plant traits								Soil traits			
		
	DBH	CT_lf_	CT_rt_	Lignin_lf_	Lignin_rt_	N_lf_	P_lf_	Lig:N_lf_	pH	C	N	C:N
Genotype												
χ^2^	13.48	10.90	6.06	34.52	1.90	0.65	15.29	11.69	3.15	8.17	18.64	24.92
*P*	**0.009**	**0.02**	0.20	**<0.001**	0.85	0.96	**0.004**	**0.019**	0.53	0.09	**<0.001**	**<0.001**
Environment												
χ^2^	18.09	14.27	15.12	46.49	5.81	21.11	10.17	28.60	104.73	133.34	101.95	85.57
*P*	**<0.001**	**<0.001**	**<0.001**	**<0.001**	0.055	**<0.001**	**0.006**	**<0.001**	**<0.001**	**<0.001**	**<0.001**	
G × E												
χ^2^	30.05	17.03	9.67	11.54	4.06	7.69	6.86	4.45	17.22	14.01	7.84	18.01
*P*	**<0.001**	**0.029**	0.29	0.17	0.85	0.46	0.55	0.81	**0.028**	0.08	0.45	**0.021**

Plant traits include tree diameter at breast height (DBH; in cm) and the concentration of condensed tannins (CT), lignin, nitrogen (N), phosphorus (P), and the ratio of lignin:N for leaf litter and/or root tissues. Soil traits include soil pH, soil carbon (C), soil nitrogen (N) and the ratio of soil C:N. All variables, except leaf and root lignin and leaf condensed tannins were log +1 transformed. Significant values (α = 0.05) are presented in bold.

**Figure 2:**
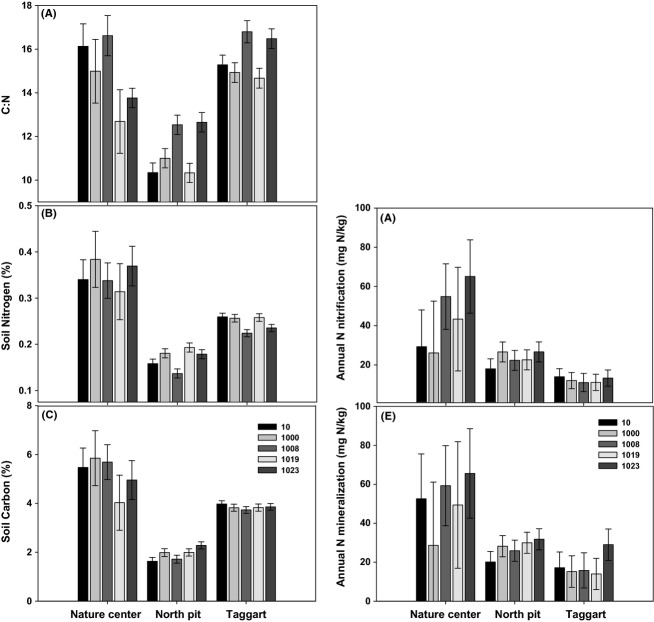
Role of plant genotype (G), environment (E), and G × E interactions on soil carbon (C) and nitrogen (N) pools, annual rates of net N mineralization and net nitrification. Mean organic soil C (A), total N (B), soil C:N (C), annual rates of net nitrification (D), and net N mineralization (E) averaged from soils beneath five *Populus angustifolia* genotypes reciprocally planted in three common gardens in northern Utah. (*n* = 3–5 replicates of each genotype/garden). Each bar represents a different genotype ±1 SE. See Tables 2 and 3 for statistical results.

Total soil N and C:N ratio also varied by genotype while soil C was not significantly different across genotypes (Table 2). Annual rates of net N mineralization and net ammonification (figure not shown) did not vary significantly by genotype, however, there was a significant response of net nitrification among plant genotypes. Across all genotypes, the greatest annual rates of net N mineralization were found at the low elevation garden (54.0 mg N/kg), and these rates were nearly three times higher than the rates found at the high elevation garden (18.19 mg N/kg) (Fig. 2E). Soil pH, known to influence microbial communities, did not vary significantly by genotype (Tables 2) but did vary significantly by common garden environment (*P* < 0.001) as well as demonstrating a significant G × E interaction (*P* = 0.028).

### Environment and G × E interactions

Consistent with our hypothesis that the effects of genetically based plant–soil linkages will vary by environment, we found that tree phenotypes varied across common garden environments. Trees at the lowest elevation common garden (i.e., Nature Center) had the greatest DBH, and were 9.5–11.5 cm greater on average than the two higher elevation gardens. There were also significant differences in tissue quality across common garden environments. Leaf litter CT, lignin, N, P, and lignin:N ratios all varied across the three environments, while roots only varied in CT (Table 2). The highest leaf lignin was found at the low elevation site, the lowest leaf lignin was found at the highest elevation site (i.e., Taggart); the intermediate site was not significantly different than the low elevation site (Table 2). We found significant G × E interaction for DBH and leaf CT but no significant G × E interactions (Table 2) for leaf lignin or N and P tissue quality indicating that the mean genotype values (especially for leaf litter traits) were stable across environments.

Nutrient pools measured as soil organic C (% C), total N (%N), and C:N ratio were found to vary significantly by common garden environment. There were also a strong environmental influence over soil nitrogen dynamics including net N mineralization, net ammonification, and net nitrification (Table 3, Fig. 2D and E). Moreover, we found that soil pH and C:N ratio demonstrated a G × E interaction whereby the rank order of the genotype means changed across environments (Tables 1 and 3, Fig. 2). There were also significant G × E interactions for net N mineralization and nitrification (Table 3, Fig. 2).

**Table 3:** Repeated measures results (MANOVA) showing annual rates of soil net nitrogen mineralization (N_min_), net ammmonification and net nitrification rates under *Populus angustifolia* genotypes grown in three common gardens in northern Utah

	N_min_		Ammonification		Nitrification	
			
	*F*	*P*	*F*	*P*	*F*	*P*
Time	17.15	<0.001*	10.47	<0.001*	29.98	<0.001*
Time × Genotype (G)	1.16	0.32	1.55	0.12	2.54	0.005*
Time × Environment (E)	5.99	<0.001*	3.55	0.003*	5.29	<0.001*
Time × G × E	1.92	0.011*	1.21	0.24	2.83	<0.001*

Significant values (α = 0.05) are presented in bold and with an asterisks.

### Plant–Soil linkages

The significant impacts of plant genetic variation and their interaction with the environment (G × E) varied among specific plant and soil traits, however, environment had strong impacts on all plant and soil traits (Table 2, Fig. 2). Soil N and C varied by over 50% and 62% across all common environments (Table 2, Fig. 2A–C); there was no G × E interaction effect, suggesting that phenotypic plasticity in plant traits help regulate soil nutrient dynamics over the life of the tree. Within each of the common garden environments, soil nutrient processes (N_min_) were negatively related to leaf lignin content with the regression (data not shown). No environment × leaf lignin interaction was detected with ANOVA indicating that the effects of leaf lignin varied subtly across all common gardens (Fig. 1C). In a subsequent multiple regression analysis examining the phenotypic correlation, across all environments, there was no significant relationship between annual rates of net N mineralization and any of the measured plant traits suggesting that the regulation of net N mineralization by lignin concentration varies by environment (results not shown). In contrast to leaf lignin, tree DBH was positively correlated to soil total C and N pools were related to tree DBH (Table 4) within each of the common garden environments, there was no common garden × tree DBH interactions indicating that the effects of tree DBH were consistent across all environments. Multiple regression analysis examining the phenotypic correlation among plant traits and soil N, across all sites, largely confirm the ANCOVA results (Table 4). Total soil N was significantly related to tree DBH and foliar CT (DBH, *F* = 20.99, *P* < 0.0001; leaf CT, *F* = 5.47, *P* = 0.023). Total soil C was significantly related to tree DBH and foliar CT (DBH, *F* = 23.40, *P* = <0.001; leaf CT, *F* = 12.59, *P* = 0.008). These results suggest genetic-based plant–soil feedbacks occur whereby phenotypic expression in plant growth and leaf litter traits are the primary biotic mechanisms driving the soil nutrient pools.

**Table 4:** ANCOVA results indicating correlations between plant traits and soil nitrogen (N) and carbon (C) pools and N processes (annual rates of net N mineralization [N_min_]) associated with five *Populus angustifolia* genotypes within three common gardens (Environment) in northern Utah. (site)

Model	Soil N (%)			Soil C (%)			N_min_ (mg N/kg)		
		
	*F*_ratio_	*P*	*r*^2^	*F*_ratio_	*P*	*r*^2^	*F*_ratio_	*P*	*r*^2^
Environment	24.47	<0.001*	0.80	20.90	<0.001*	0.85	8.90	<0.001*	0.50
Leaf lignin	0.006	0.94		0.30	0.58		6.68	0.013*	
DBH	6.07	0.017*		5.41	0.02*		0.73	0.40	
Leaf tannin	0.53	0.47		0.85	0.36		0.41	0.71	
Environment × Leaf lignin	0.58	0.56		0.48	0.62		1.50	0.23	
Environment × DBH	1.27	0.29		1.30	0.28		1.02	0.37	
Environment × Leaf tannin	0.002	0.99		1.54	0.23		1.36	0.37	

Significant values (α = 0.05) are presented in bold and with an asterisks.

## Discussion

Overall, these results indicate that phenotypically plastic plant traits along an elevation gradient alter soil nutrient pools and processes. We found that soil C and N pools and process rates are intimately linked to plant phenotypes (growth and litter chemistry), which can be influenced by inherent genetic differences within a tree species, their environment, and the interaction of the two. Moreover the results show that phenotypic responses can be the drivers in these plant and soil interactions. Within and across common garden sites, tree growth was positively related to total soil N and C. These results suggest that genetically based differences in growth (Lojewski et al. 2009, 2012) may result in an internal positive feedback whereby biomass accumulation underneath specific genotypes improves the nutrient environment of trees at local and landscape scales (i.e., genotype-based soil niche construction occurs; sensu Pregitzer et al. 2010; Smith et al. 2012).

### Genetic by environment interactions

Overall, these results show that genotype, environment and, in two cases, their interaction drive variation in plant traits that can regulate soil nutrient pools and processes. In particular, plant growth (i.e., DBH) appears to be important at local and landscape scales. It is well established that plant growth and other factors such as leaf chemistry have a genetic basis that can be influenced by biotic and abiotic factors including soil microbial communities, herbivory, fertilization, and shading, for example (Lindroth et al. 2002; Rehill et al. 2006; Lojewski et al. 2009, 2012; Madritch and Lindroth 2011). These results are largely supportive of those predictions. The only case where we found no genetic or environmental effect was in root lignin concentration. For foliar CT and tree DBH, we found significant G × E interactions in those traits. Additionally for tree DBH, this interaction suggests that site-specific environmental factors that were not measured could play an important role. For example, DBH is greatest at the high elevation site, intermediate at the low elevation site, and lowest at the intermediate elevation site (Fig. 2). While some environmental factors such as temperature decrease along an elevational gradient site specific differences such as localized temperature or precipitation differences could play a role in impacting plant phenotype.

It has been recognized that genetic variation in one species can have extended effects at the community and ecosystem level (Schweitzer et al. 2004, 2012b; Madritch et al. 2006, 2009; Whitham et al. 2006; Johnson and Stinchcombe 2007; Hughes et al. 2008). Less is understood how genetic variation interacts with environmental variation to alter ecosystem function and whether the effects of genetic variation at the ecosystem level are largely local phenomena relative to other environmental factors across the landscape. This study indicates that plant genotype and environmental variation impact plant–soil linkages, through genotype-specific responses in plant phenotypes (DBH, CT_lf_), to affect soil N and C pools and annual rates of net N mineralization. Studies experimentally examining the relative effects of genetic versus environment on ecosystem processes have predictably found strong effects of environmental variation (primarily via fertilization) in mediating the ecosystem consequences of genotype (Madritch et al. 2006; Boydon et al. 2008). Our study is one of the first to examine the relative effects of genetic versus environment in a natural system and our results show the strength of the plant–soil feedback under a range of natural environments and the importance of genetic variation in the field. While we found environment plays a strong role in mediating plant phenotypes (i.e., genotypes demonstrate phenotypic plasticity), changes in plant phenotype affect soil nutrient pools and extended effects of plant genotype.

The links we found in above- and belowground responses from this study parallels other studies where variation in above- and belowground tissue quality and productivity among species or functional groups can cause shifts in C and N pools and fluxes (Hobbie 1992; Tilman et al. 1997; Binkley and Giradina 1998; Finzi et al. 1998; Ehrenfeld 2003). Because we utilized three common gardens in this study we attribute the G × E effects of net N_min_ to variation in plant phenotypes regulated by the genetic response to the different environments. Moreover, because the common gardens exist along an elevational gradient changes in the phenotypic rank of individual genotypes could suggest that some genotypes are more responsive to environmental variation than others. Such results suggest that there may be plasticity in the ability of plants to regulate their soil environment through plant–soil feedbacks. Such plasticity in the ability to regulate nutrient process rates may be due to the response of the soil microbial community across substrates and environments. For example, temperature and moisture changes can shift the microbial community composition to alter the rates of soil organic matter decomposition (Zogg et al. 1997; Waldrop and Firestone 2006). Also, in the *Populus* system, genetically based substrate inputs from tree genotype have been shown to predict the soil microbial community composition (Schweitzer et al. 2008), to the extent that when litter from tree genotypes are exchanged and placed beneath other genotypes, the microbial community conforms to the new litter environment (Madritch and Lindroth 2011). Because genetic and environmental effects on the pools of organic total N and C in the soil and G × E interactions for C:N ratio were found, these results suggest that there are tight links from the plant phenotype (DBH, CT_lf_) to soil C and N pools (Table 4).

While links between plants and soils are apparent, the mechanisms contributing to the variation we see in plant phenotype, soil phenotype, and soil processes are more complex. Our results show that DBH and litter chemistry (leaf lignin) are related to increased net N mineralization and total soil N and C at local and landscape scales. Additionally, higher soil N typically results in greater growth (LeBauer and Treseder 2008) which provides evidence of a genetically based positive feedback where increased plant growth from phenotypic plasticity will improve the nutrient environment of those trees at local and landscape scales. Moreover, within *Populus* spp. recent work has show the genetic basis to tree productivity and belowground C allocation (Lojewski et al. 2009, 2012), indicating that genetic-based differences in growth can have extended consequences for soil processes. However, nutrient pools and processes are mediated by the microbial community that is likely to vary by environment, which make it difficult to disentangle which factors are driving plasticity in these ecosystem level responses. This is important as differences in nutrient processing in soil beneath different tree genotypes can also have fitness consequences in the next generation. For example, in this same system it was found that a positive plant–soil feedback occurs where *P. angustifolia* genetic families had fitness advantages (twice as likely to survive, grew 24% taller, had 27% more leaves, and 29% greater aboveground biomass) when grown in soil collected from beneath other *P. angustifolia* trees versus soils collected from other *Populus* species and hybrids in the same river drainage (Pregitzer et al. 2010). Smith et al. (2012) found a similar positive feedback when *P. angustifolia* families were planted in soils collected beneath the trees where the seeds were collected relative to soils associated with other trees. Plant–soil feedbacks are just beginning to be placed in an evolutionary perspective (Pregitzer et al. 2010; Lankau et al. 2011; Schweitzer et al. 2012b) and have shown that soil legacy effects due to genetically mediated plant–soil feedbacks may promote the success of some genotypes over others leading to the creation of spatial forest mosaics across landscapes (Thompson 2005; Madritch et al. 2009).

## Conclusions and Implications

These results overall show that the environment has strong effects on plant phenotypes and that responses (i.e., in plant growth and litter chemical traits) can be correlated with leaf litter inputs to soils that can influence belowground pools and process rates. These data further suggest that genotypes may contribute to total C and N pools at local and landscape scales. As we make predictions for future ecosystems, G × E studies may help us understand the factors influencing plant–soil feedbacks, the plasticity of genotypes within a species, and the potential fitness and evolutionary consequences of plant–soil linkages in forest stands across environments and landscapes. With changes in the global climate, these types of studies indicate that higher elevation trees that experience temperatures outside their natural range of variability may experience potential shifts in net N mineralization and nutrient pools. For example, within our common gardens on average, there is a 3°C difference between our low elevation and high elevation (7.7°C at Taggart and 10.7°C at Nature Center), therefore based on our results we could predict two- to fivefold shifts in C storage and rates of net N mineralization given an increase in 3°C (i.e., range of responses from lowest to highest elevation sites). Understanding the community and ecosystem consequences of such dynamics represents a fundamental and yet largely unexplored area of research in predicting the landscape level consequences of global change factors.
